# Effects of self-help mindfulness-based cognitive therapy on mindfulness, symptom change, and suicidal ideation in patients with depression: a randomized controlled study

**DOI:** 10.3389/fpsyg.2023.1287891

**Published:** 2023-12-01

**Authors:** Yuanyuan Mo, Zhiying Lei, Mei Chen, Hongyan Deng, Rong Liang, Miaoyu Yu, Huiqiao Huang

**Affiliations:** ^1^Department of Psychiatry, The Second Affiliated Hospital of Guangxi Medical University, Nanning, China; ^2^Department of Nursing, The Second Affiliated Hospital of Guangxi Medical University, Nanning, China; ^3^Department of Nursing, Dongguan People’s Hospital, The Tenth Affiliated Hospital of Southern Medical University, Dongguan, China

**Keywords:** self-help mindfulness-based cognitive therapy, depression, mindfulness, suicidal ideation, randomized controlled study

## Abstract

**Objective:**

This study aimed to evaluate the effects of self-help mindfulness-based cognitive therapy (MBCT-SH) on mindfulness, symptom change, and suicidal ideation in patients with depression.

**Methods:**

For this randomized controlled study, 97 patients were randomly assigned to either the MBCT-SH (*n* = 48) or control (*n* = 49) group. The Five Facet Mindfulness Questionnaire (FFMQ), Hamilton Depression Rating Scale (HAMD-24), and Suicide Attitude Questionnaire (SAQ) were used to assess mindfulness, depression symptoms, and suicidal ideation, respectively, at baseline (T0), intervention week 4 (T1), intervention week 8 (T2), and 3-month follow-up (T3). The groups were also compared on treatment costs and readmission rates at a 6-month follow-up.

**Results:**

In the MBCT-SH group, 46 of 48 participants (96%) completed the eight-week program. At T0, there were no statistically significant between-group differences in demographics, clinical characteristics, FFMQ, HAMD-24, or SAQ. Nor were there statistically significant differences on the HAMD-24 or SAQ between the MBCT-SH and control groups at T1 (*p* = 0.18 and *p* = 0.59, respectively), while mindfulness was significantly higher in the MBCT-SH group (*t* = 2.383, *p* = 0.019). At T2, there were significant between-group differences on the FFMQ, HAMD-24, and SAQ, all of which remained significant at T3. At the 6-month follow-up, *per capita* treatment costs were 5,298 RMB lower in the MBCT-SH group compared with the control group, while their readmission rates (6.1% and 4.2%, respectively) did not differ significantly.

**Conclusion:**

These findings support the feasibility and effectiveness of MBCT-SH among patients with depression.

**Clinical trial registration:**

http://www.chictr.org.cn, ChiCTR2300077850.

## 1 Introduction

Depression is a mental illness characterized by low mood, sadness, and a sense of emptiness (American Psychiatric Association, 1980) that affects about 280 million people worldwide (Ortega et al., 2022) and contributes greatly to the global disease burden. In China, the lifetime prevalence of depression is 6.9%, and the 12-month prevalence is 3.6% (Huang et al., 2019). Depression is distinct from typical mood fluctuations and short-term emotional responses to everyday challenges. Especially when recurrent, moderate or severe depression can become a serious health issue that causes patient suffering and impacts their work, school and relationship functioning.

At its worst, depression can lead to suicide. Among completed suicides, 43–50% can be attributed to depression (Slama et al., 2011), and depression causes one of the highest suicide rates among psychiatric disorders. These patients see suicidal behavior as a solution, to escape psychological pain (Campos et al., 2017). Clinical depression treatment focuses primarily on pharmacotherapy, which can improve clinical symptoms and reduce suicide incidence. Yet suicide risk may remain during antidepressant treatment, and some patients have residual symptoms; thus, there are limitations to single drug therapy (Yuan et al., 2020). In recent years, patients with depression have also benefited from concurrent physical therapy and psychotherapy. However, even with a variety of treatments, some patients have persistent symptoms and do not fully recover (Pigott et al., 2010). Therefore, optimal depression treatments still need to be explored.

Mindfulness-based cognitive therapy (MBCT) was first proposed in 2003 (Morgan, 2003) as a novel approach to preventing depression relapse. MBCT is typically an eight-week group intervention of mindfulness-based stress reduction and cognitive behavioral therapy (Wright and Beck, 1983; Kabat-Zinn, 1990). Multiple studies have shown that MBCT can significantly reduce both current depression symptoms and relapse risk and related symptoms in patients who are in remission from depression (Cladder-Micus et al., 2018; Zemestani and Fazeli, 2020; Wang et al., 2022). While MBCT can be beneficial for depression recovery, several factors have limited its implementation. First, available space can limit group size. Second, time demands can reduce participation rates. Third, intensive face-to-face training may increase negative emotions in patients with depression. Thus, novel ways to address these limitations, and facilitate more patients with depression who can benefit from MBCT, are needed.

Self-help MBCT (MBCT-SH) is an internet-based intervention in which participants conduct self-help mindfulness training with a mobile device (e.g., smartphone, computer) and audio-visual materials. While self-help mindfulness interventions have been confirmed as efficacious (Zernicke et al., 2013; Lever Taylor et al., 2014; Kvillemo et al., 2016), and online MBCT-SH is attracting increasing attention, it is still in the exploratory stage. Few studies have evaluated the effectiveness of MBCT-SH in patients with depression. As such, the aim of this randomized controlled study was to determine the effects of MBCT-SH on mindfulness, symptom change, and suicidal ideation in patients with depression.

## 2 Materials and methods

### 2.1 Study design

**Figure 1:**
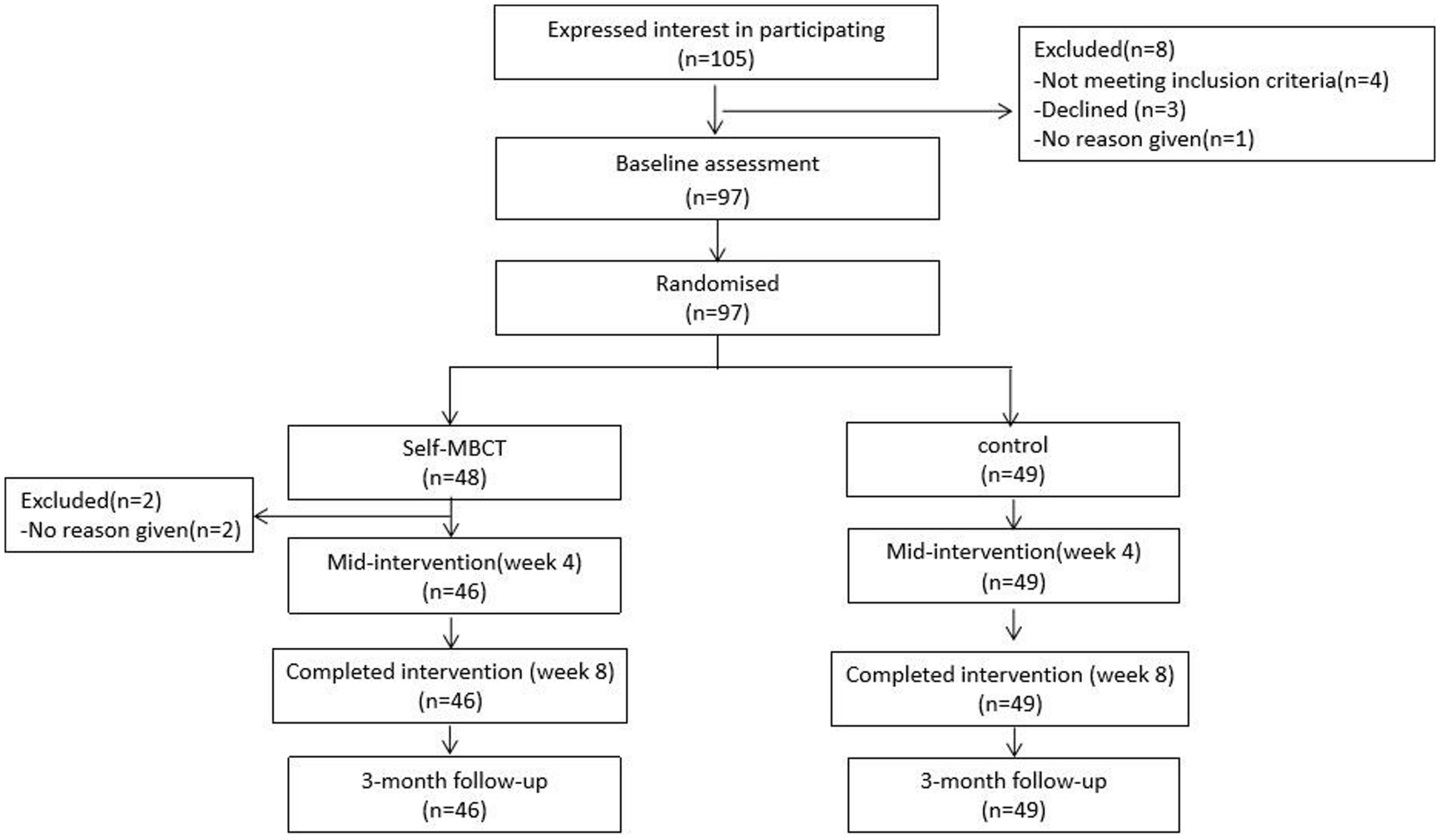
CONSORT diagram.

As illustrated by Figure 1, for this randomized controlled trial comparing MBCT-SH and control groups of Chinese patients with depression, participants were assessed at baseline (T0), intervention week 4 (T1), intervention week 8 (T2), and 3-month follow-up (T3).

### 2.2 Study sample and protocol

#### 2.2.1 Sample size

Power was calculated using G*Power (Faul et al., 2007); with *p* = 0.05 and power 80%, 86 participants were needed to detect effect sizes comparable to those reported in a similar mindfulness-based intervention study (Perez-Blasco et al., 2013). Accounting for a potential 10% attrition during follow-up, we sought to include at least 95 participants.

#### 2.2.2 Inclusion and exclusion criteria

Inclusion criteria were: depression diagnosis based on associate chief- and psychiatrist-evaluated DSM-IV diagnostic criteria, through administration of the Chinese version of the mini international neuropsychiatric interview; age 18–60 years; total Hamilton Depression Rating Scale (HAMD-24) score ≥ 8; ability to communicate in Chinese; normal reading and communication abilities; able to sign written informed consent.

Exclusion criteria were: meeting the DSM-IV Axis I disorder diagnostic criteria for another psychiatric disorder; suffering from a severe physical disease, central nervous system disease, or substance abuse; inability to use a smartphone or computer; already regularly practice mindfulness meditation.

#### 2.2.3 Participant recruitment

Participants were recruited from the psychological counseling clinic, psychiatry clinic, and psychiatric inpatient department at the Second Affiliated Hospital of Guangxi Medical University. Potential participants were informed about the study and signed informed consent. The study was conducted from June 2021 to December 2022.

#### 2.2.4 Group assignment and intervention setting

Participants were randomly assigned at a 1:1 ratio to either the MBCT-SH or control group. Patients in both groups received conventional therapy; patients in the MBCT-SH group also received the eight-week MBCT-SH program.

#### 2.2.5 Self-help mindfulness-based cognitive therapy group

The intervention was implemented according to the translated MBCT manual for depression (Morgan, 2003). Prior to training, participants in the MBCT-SH group were trained in the use of mindfulness exercises, including the participant manual and MBCT audio exercises. The participant manual included the MBCT definition, weekly training schedule, exercise instructions, and information about commitment to the training program. MBCT-SH group participants were also trained to use the audio and manual by MBCT-certified instructors with extensive experience in mindfulness training.

Participants were encouraged to practice 30–45 min daily, 3–5 days per week. The eight-week MBCT-SH program is shown in Table 1. A researcher also introduced the daily training content and following week’s training project through a WeChat group every Monday. Participants needed to be willing to record and share their feelings about the program through WeChat, email, or phone.

**Table 1:** MBCT-SH program content.

	Topic	Psychoeducation	Exercise
Week 1	Waking up from automatic guided	Psychological reactions of patients with depression	Mindfulness eating
Week 2	Returning to present experiences	Association of mood and thoughts	Body scan/Pleasant activity and event record
Week 3	Concentrating the scattered mind	Pleasant activities and events	Gentle yoga/Mindful walking
Week 4	Staying present	Reactions to pleasant and unpleasant events	Mindfulness meditations/Three-minute breathing space exercise
Week 5	Letting it be	Acceptance	Building pleasant habits/Record of appreciation and gratitude/Mindfulness meditations
Week 6	Thoughts are not facts	Cognition	Record of appreciation and gratitude/Mindfulness meditations
Week 7	Better self-care	Choosing functional behaviors	Body scan/Record of appreciation and gratitude
Week 8	Using what you learn to deal with the future	Plans for future practice	/

#### 2.2.6 Control group

Control group participants received conventional therapy only. They were disallowed from participating in any type of mindfulness program during the study. They were invited to attend the MBCT-SH program upon completion of their study participation.

### 2.3 Assessments

#### 2.3.1 Hamilton depression scale-24

The HAMD-24, developed by Hamilton in 1960 (Hamilton, 1960), is widely used to evaluate depression symptom severity in adults. The 24 items are each rated on a four-point scale, and total scores are interpreted as follows: ≤7, no depression; >8 to ≤20, mild depression; >20 to ≤35, moderate depression; and > 35, severe depression.

#### 2.3.2 Suicide attitude questionnaire

The Suicide Attitude Questionnaire (SAQ), developed by Xiao et al. (1999), is used to evaluate attitudes and views towards suicide. The scale consists of four dimensions: understanding the nature of suicidal behavior; attitude toward suicide; attitude toward the family of the suicide; and attitude toward euthanasia. SAQ scores are interpreted as follows: ≤2.5, positive attitude toward suicide; >2.5 to <3.5, ambivalence or neutrality; and ≥ 3.5, negative attitude toward suicide.

#### 2.3.3 Five facet mindfulness questionnaire

The Five Facet Mindfulness Questionnaire (FFMQ), developed by Baer et al. (2006), includes: observing; describing; non-judging of inner experience; acting with awareness; and non-reactivity to inner experience. Each of the 39 items are rated on a five-point scale.

### 2.4 Data analysis

SPSS 24.0 software was used for data analyses. Descriptive statistics are used to describe the participant characteristics and assess levels of depression, suicide attitude, and mindfulness. Continuous variables were analyzed by *t*-test and categorical variables by χ^2^ test, to test for between-group differences at T0. Between-group comparison measures were first assessed for normality. Data conforming to the normal distribution were tested by independent samples *t*-tests. Paired samples *t*-tests were used to compare within-groups, and repeated measures ANOVAs were used to test for changes in indices. *p* < 0.05 was considered statistically significant.

## 3 Results

### 3.1 Participation rate

Ninety-seven of 105 potential participants signed informed consent and 8 were excluded. The 97 participants were randomized to the MBCT-SH (*n* = 48) or control (*n* = 49) group.

### 3.2 General data

The average participant age was 30.06 (standard deviation [SD] = 11.82) years. The participants were overwhelmingly female, and more than half were college-educated. Most had moderate depression. At T0, there were no statistically significant between-groups differences in demographic or clinical characteristics (Table 2).

**Table 2:** Group demographic and clinical characteristics.

	MBCT-SH group (*n* = 48)	Control group (*n* = 49)	t/χ^2^	*p*
**Age (years, mean)**	31.02 (SD = 12.80)	29.12 (SD = 10.83)	0.789	0.432
**Gender (%)**			0.874	0.350
Male	18 (37.50)	14 (28.60)		
Female	30 (62.50)	35 (71.40)		
**Marriage (%)**			1.376	0.503
Married	23 (47.90)	23 (46.90)		
Single	20 (41.70)	17 (34.70)		
Divorced or other	5 (10.40)	9 (18.40)		
**Education (%)**			0.281	0.596
High school or below	19 (39.60)	22 (44.90)		
Junior college or higher	29 (60.40)	27 (55.10)		
**Duration of diagnosis (%)**			0.600	0.963
Within 3 months	11 (22.90)	13 (26.50)		
3–6 months	6 (12.50)	8 (16.30)		
˃6 months to <12 months	8 (16.70)	7 (14.30)		
1–3 years	11 (22.90)	10 (20.40)		
>3 years	12 (25.0)	11 (22.40)		

### 3.3 Five facet mindfulness questionnaire

**Figure 2:**
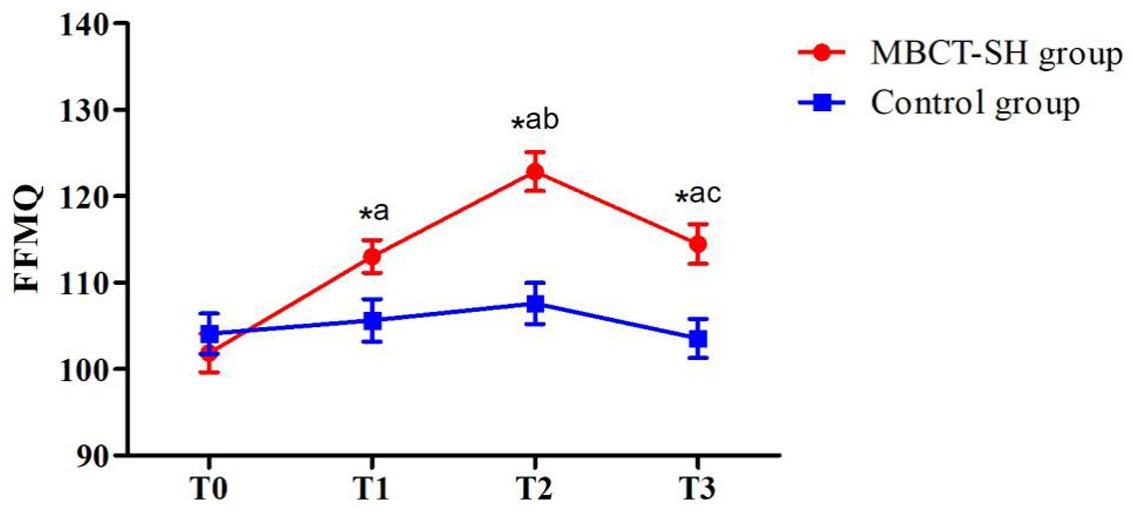
Mean FFMQ scores from T0 to T3 in MBCT-SH and control groups. ^a^*p* < 0.05 vs. T0; ^b^*p* < 0.05 vs. T1; ^c^*p* < 0.05 vs. T2; FFMQ, five-facets of mindfulness.

As shown in Figure 2 and Table 3, repeated measures ANOVA showed significant main FFMQ effects of group (*F* = 7.905, *p* = 0.006) and time (*F* = 30.054, *p* < 0.001). There was also a significant interaction between group and time (*F* = 16.657, *p* < 0.001). Therefore, simple effects analyses were carried out, comparing groups based on time. There was not a significant between-groups difference at T0, indicating comparability at baseline. However, between-groups differences at T1, T2, and T3 were statistically significant, with higher MBCT-SH group scores compared with control group scores. Within-group comparisons showed that the MBCT-SH group differed significantly between T1, T2, and T3 compared with T0, and between T1 and T3 compared with T2. The control group did not differ significantly across any time points.

**Table 3:** FFMQ scores for MBCT-SH and control groups.

	Group	*n*	T0	T1	T2	T3	*F,p*		
							Group	Time	Interaction
FFMQ	MBCT-SH	48	101.88 ± 15.36	113.04 ± 13.03^a^	122.85 ± 15.56^ab^	114.48 ± 16.01^ac^	*F* = 7.905, *p* = 0.006	*F* = 30.054, *p* < 0.001	*F* = 16.657, *p* < 0.001
	Control	49	104.10 ± 16.26	105.65 ± 17.17	107.59 ± 16.58	103.57 ± 15.77			
	*t*		−0.693	2.383	4.673	3.33			
	*P*		0.49	0.019	<0.001	0.001			

^a^*p* < 0.05 vs T0; ^b^*p* < 0.05 vs T1; ^c^*p* < 0.05 vs T2. FFMQ, five facet mindfulness questionnaire.

### 3.4 Hamilton depression rating scale

**Figure 3:**
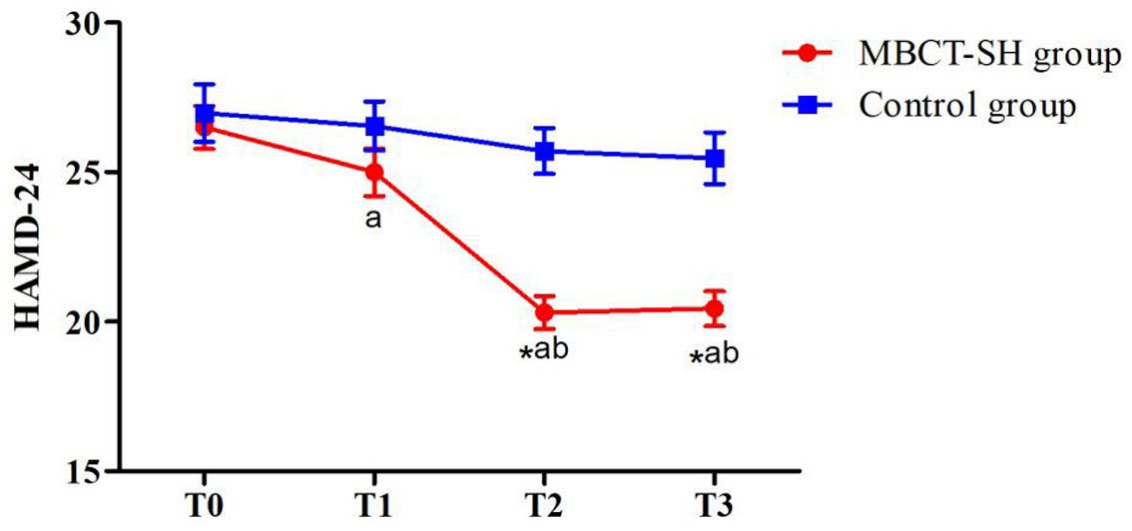
Mean HAMD-24 scores from T0 to T3 in MBCT-SH and control groups. ^a^*p* < 0.05 vs. T0; ^b^*p* < 0.05 vs. T1; ^c^*p* < 0.05 vs. T2; HAMD-24, Hamilton depression scale-24.

There were significant HAMD-24 score difference effects for group (*F* = 11.011, *p* = 0.001) and time (*F* = 36.812, *p*<0.001). There was also an interaction between group and time (*F* = 15.050, *p*<0.001). Therefore, simple effects analyses were conducted, comparing between-group on time. As shown in Figure 3 and Table 4, there was no significant between-groups difference at T0, indicating that the groups were comparable. Nor was there a significant between-groups difference at T1. At T2 and T3, MBCT-SH group scores were significantly lower compared with the control group. Within-groups comparisons showed that the MBCT-SH group had significant differences between T1, T2, and T3 compared with T0, and between T2 and T3 compared with T1. There were no significant control group differences across any time points.

**Table 4:** HAMD-24 scores for MBCT-SH and control groups.

	Group	*n*	T0	T1	T2	T3	*F,p*		
							Group	Time	Interaction
HAMD-24	MBCT-SH	48	26.50 ± 4.98	25.00 ± 5.54^a^	20.31 ± 3.82^ab^	20.44 ± 3.40^ab^	*F* = 11.011, *P* = 0.001	*F* = 36.812, *p* < 0.001	*F* = 15.050, *p* < 0.001
	Control	49	26.98 ± 6.70	26.55 ± 5.76	25.71 ± 5.40	25.47 ± 6.02			
	*t*		−0.400	−1.351	−5.681	−4.844			
	*P*		0.690	0.180	<0.001	<0.001			

^a^*p* < 0.05 vs T0; ^b^*p* < 0.05 vs T1; ^c^*p* < 0.05 vs T2. HAMD-24, Hamilton depression scale-24.

### 3.5 Suicide attitude questionnaire

**Figure 4:**
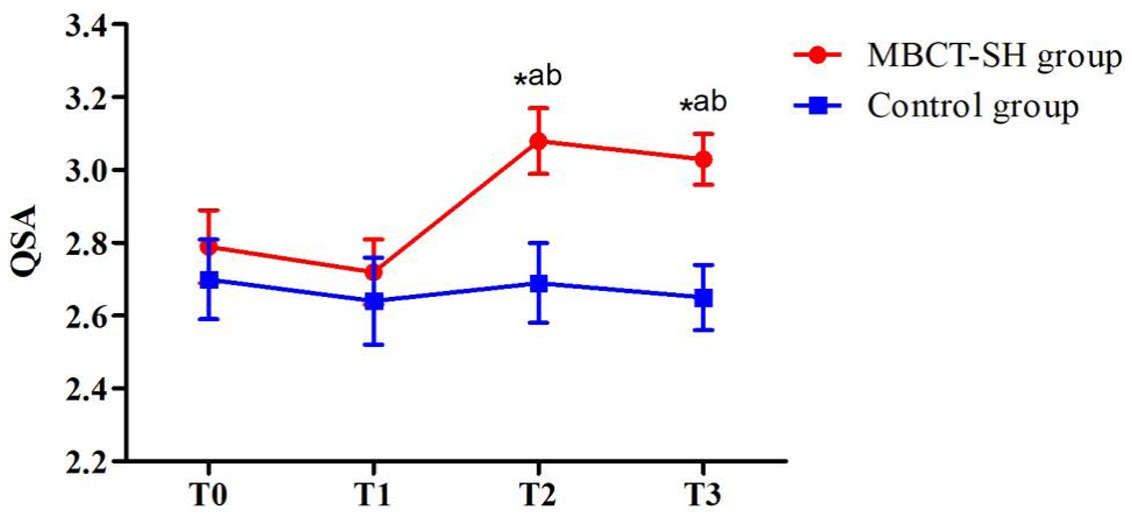
Mean SAQ scores from T0 to T3 in MBCT-SH and control groups. ^a^*p* < 0.05 vs. T0; ^b^*p* < 0.05 vs. T1; ^c^*p* < 0.05 vs. T2; SAQ, suicide attitude questionnaire.

As shown in Figure 4 and Table 5, repeated measures ANOVA showed significant main SAQ effects for group (*F* = 3.433, *p* = 0.067) and time (*F* = 8.937, *p* < 0.001). There was also an interaction between group and time (*F* = 7.968, *p* < 0.001). Therefore, simple effects analyses were carried out, comparing between groups based on the time factor. There was not a significant between-groups difference at T0, indicating that the groups were comparable at baseline. Nor was there a significant between-groups difference at T1. However, the groups differed significantly at T2 and T3, when MBCT-SH group scores were significantly lower compared with the control group. Within-group comparisons showed that the MBCT-SH group differed significantly at T2 and T3 compared with T0, and between T2 and T3 compared with T1. The control group showed no significant differences across time points.

**Table 5:** SAQ scores for MBCT-SH and control groups.

	Group	*n*	T0	T1	T2	T3	*F,p*		
							Group	Time	Interaction
SAQ	MBCT-SH	48	2.79 ± 0.67	2.72 ± 0.63	3.08 ± 0.60^ab^	3.03 ± 0.49^ab^	*F* = 3.433, *p* = 0.067	*F* = 8.937, *p* < 0.001	*F* = 7.968, *p* < 0.001
	Control	49	2.70 ± 0.80	2.64 ± 0.81	2.69 ± 0.74	2.65 ± 0.60			
	*t*		0.568	0.541	2.876	3.387			
	*p*		0.571	0.590	0.005	0.001			

^a^*p* < 0.05 vs T0; ^b^*p* < 0.05 vs T1; ^c^*p* < 0.05 vs T2. SAQ, suicide attitude questionnaire.

We also compared treatment costs and readmission rates between the groups at a 6-month follow-up. At that time, *per capita* treatment costs were 5,298 RMB lower in the MBCT-SH group compared with the control group. MBCT-SH and control group readmission rates, 6.1% and 4.2%, respectively, did not differ significantly (*p* > 0.05).

## 4 Discussion

Previous studies (Zhang et al., 2018; Yu et al., 2019) have shown that traditional MBCT intervention training reduces non-inpatient enthusiasm and compliance, because of inflexible meeting times and locations. This study tested a novel self-help internet-based MBCT training program, in which patients could flexibly adjust training content and timing according to their needs, and without affecting their daily life. Self-help training can also reduce patient financial burden by lowering treatment costs, transportation costs, lost income, and other expenses.

Herein, 46 of 48 participants (96%) completed both the four-week MBCT self-help program and eight-week follow-up. Participants also showed a high level of adherence to MBCT-SH, similar to traditional MBCT (Cavanagh et al., 2013). Participants herein actively accepted MBCT-SH, perhaps due to its accessibility, and possibly because 62.50% of the sample was female. Previous studies have shown that females are more interested in participating in mindfulness training programs (Bränström et al., 2012). To the best of our knowledge, few studies have assessed self-help MBCT in patients with depression. In a similar study of self-help MBCT in students, the participation rate was 95% (Lever Taylor et al., 2014), close to that herein.

Herein, control group participants received conventional therapy, while the MBCT-SH group received an additional eight weeks of MBCT-SH training. As shown in Table 3, MBCT-SH group FFMQ scores were significantly higher than the control group at T1, T2, and T3 (all *p* < 0.05). Compared with T0, mindfulness in the intervention group increased by mid-intervention (T1) and was higher post-intervention (T2). This indicates that patients with depression can gradually form a stronger sense of mindfulness through MBCT-SH training. Furthermore, longer training leads to stronger mindfulness. Previous studies have shown that mindfulness levels among patients with depression rise significantly after MBCT training (Key et al., 2017; Shenefelt, 2018). However, the levels herein were slightly lower than in similar studies (Kong et al., 2020). This may have been because MBCT herein was self-help rather than traditional, thus participants could independently determine their training intensity and the researcher did not intervene overly.

There were significant between-group HAMD-24 score differences at T1, T2, and T3 (all *p* < 0.05). After eight weeks of MBCT-SH training, participants’ depression symptoms decreased from moderate to mild. This suggests that MBCT-SH may reduce depression symptoms, similar to traditional MBCT (Joormann and Davanzato, 2010; Peng and Pang, 2018). Our findings also suggest that depression symptom severity and mindfulness are inversely related. A primary psychological characteristic of patients with depression is that they pay excessive attention to negative emotions (Leppänen, 2006). Several studies (Ives-Deliperi et al., 2013; Yang et al., 2016; Lifshitz et al., 2019) have shown that the mechanism by which MBCT improves depressive symptoms is by reducing patient attention to negative emotions through the attention control network system and emotion regulation brain areas. MBCT may both influence brain function and change brain structure (Hölzel et al., 2011). Our findings support the value of MBCT-SH for use by patients with depression.

Mindfulness can inversely predict suicide risk (Mohammadkhani et al., 2015), with higher levels of mindfulness related to lower suicide risk. In other words, mindfulness may have a protective effect by reducing suicide risk (Chesin et al., 2016). Herein, it was interesting that while no significant differences in suicidal ideation were found between the groups at mid-intervention (T1), they differed significantly both post-intervention (T2) and at later follow-up (T3). Dimidjian et al. (2014) found that the longer participants took part in MBCT training, the higher their level of mindfulness became, and the lower their suicidal ideation. Consistent with this, four weeks of MBCT-SH appeared to be insufficient. At mid-intervention, patients with depression appeared to lack sufficient mindfulness levels to reduce suicidal ideation.

Herein, MBCT-SH was effective for improving mindfulness and reducing depression symptoms and suicidal ideation. This effect was most obvious at the end of the full eight-week program and was still observed at the 3-month follow-up. Training simultaneously reduced financial burden among patients with depression.

MBCT-SH herein was an internet-based intervention in which participants self-trained in mindfulness using a mobile device (e.g., smartphone, computer), combined with audio-visual materials. The main differences between MBCT-SH and traditional MBCT are that the former lacks: (1) face-to-face classes; (2) a required specific place and time for practice; and (3) group contact among participants. The advantages of MBCT-SH thus include its lower cost, ease of access to learning content, self-regulation of practice time and space, higher level of relaxation, and independence to choose exercise content. MBCT-SH is not without disadvantages, including that the researcher cannot observe the participant’s learning response and the lack of communication among group members.

There were also several study limitations. First, three follow-up months may have been insufficient; future studies should conduct longer-term follow-up to evaluate the persistence of MBCT-SH effects. Second, the scales used to evaluate the effects of MBCT-SH were quantitative; future studies could incorporate qualitative objectives. Finally, that the sample was exclusively patients with depression may have limited the generalizability of its findings.

## 5 Conclusion

MBCT-SH improves mindfulness, depression symptoms, and suicidal ideation in patients with depression.

## Data availability statement

The raw data supporting the conclusions of this article will be made available by the authors, without undue reservation.

## Ethics statement

The studies involving humans were approved by Ethics Committee of the Second affiliated hospital of Guangxi Medical University. The studies were conducted in accordance with the local legislation and institutional requirements. The participants provided their written informed consent to participate in this study.

## Author contributions

YM: Writing – original draft. ZL: Investigation, Writing – original draft. MC: Data curation, Writing – review & editing. HD: Data curation, Investigation, Writing – review & editing. RL: Project administration, Writing – review & editing. MY: Project administration, Resources, Writing – review & editing. HH: Project administration, Writing – review & editing.
